# Isocitrate dehydrogenase gene variants in cancer and their clinical significance

**DOI:** 10.1042/BST20210277

**Published:** 2021-12-02

**Authors:** Thomas Cadoux-Hudson, Christopher J. Schofield, James S.O. McCullagh

**Affiliations:** Chemistry Research Laboratory, Department of Chemistry and the Ineos Institute for Antimicrobial Research, University of Oxford, Mansfield Road, Oxford OX1 3TA, U.K.

**Keywords:** 2-hydroxyglutarate, acute myeloid leukaemia, chemotherapy resistance, gene variants, glioma, isocitrate dehydrogenase mutations

## Abstract

Human *isocitrate dehydrogenase* (*IDH*) genes encode for the IDH1, 2 & 3 isoenzymes which catalyse the formation of 2-oxoglutarate from isocitrate and are essential for normal mammalian metabolism. Although mutations in these genes in cancer were long thought to lead to a ‘loss of function’, combined genomic and metabolomic studies led to the discovery that a common *IDH 1* mutation, present in low-grade glioma and acute myeloid leukaemia (AML), yields a variant (R132H) with a striking change of function leading to the production of (2*R*)-hydroxyglutarate (2HG) which consequently accumulates in large quantities both within and outside cells. Elevated 2HG is proposed to promote tumorigenesis, although the precise mechanism by which it does this remains uncertain. Inhibitors of R132H IDH1, and other subsequently identified cancer-linked 2HG producing IDH variants, are approved for clinical use in the treatment of chemotherapy-resistant AML, though resistance enabled by additional substitutions has emerged. In this review, we provide a current overview of cancer linked *IDH* mutations focussing on their distribution in different cancer types, the effects of substitution mutations on enzyme activity, the mode of action of recently developed inhibitors, and their relationship with emerging resistance-mediating double mutations.

## Introduction

Studies on the roles of altered genetics and metabolism have a long history in cancer research; famously, Otto Warburg [1] demonstrated increased lactate production in cancer cells grown in normoxic conditions. The exact mechanisms orchestrating the change towards glycolytic metabolism in some cancer cells have, however, remained elusive. Research in 2006 analysing cancer-associated mutations revealed an *isocitrate dehydrogenase 1* (*IDH 1*) mutation in a colorectal cancer, leading to the R132C IDH1 variant [2]. Analysis of primary brain tumours revealed a heterogenous G395A mutation in *IDH1* (R132H) in 5 of 22 glioblastoma (GBM) patients with a further 3 GBM patients having the R132S variant [3]. Interestingly, the *IDH1* mutation in primary brain tumours is linked to better survival [3]*.* The heterogeneous nature of the G395A mutation, likely resulting in heterodimeric IDH proteins, suggests that rather than a simple loss of function, the IDH1 variants may catalyse a neomorphic reaction, i.e. one not catalysed by homodimeric wild-type IDH1 (wtIDH1). Indeed, in a breakthrough study the R132H IDH1 variant was shown to enhance the production of the endogenous metabolite 2-hydroxyglutarate (2HG), which was observed to accumulate to concentrations in the range 3–35 µM/g of tumour in patient R132H IDH1 glioma tissues, whilst wild-type IDH1 tumours manifested over 100-fold less 2HG [4]. U87MG glioblastoma cells transfected with a pCMV6 plasmid encoding for R132H IDH1 also accumulated 2HG. The carbon atoms of 2HG were shown to derive from glutamate using ^13^C-tracer experiments [4].

**Figure 1.**
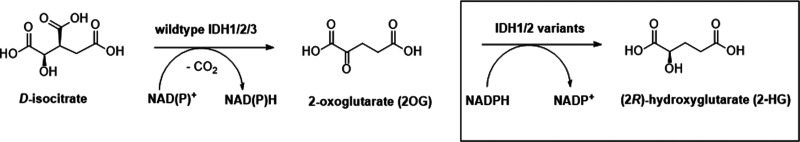
Reactions catalysed by wild-type IDH and the gain of function IDH1/2 variants. Note, IDH1 and IDH2 catalyse the reversible NADP^+^ dependent production of 2OG and CO_2_, whereas IDH3 employs NAD^+^ in an apparently irreversible reaction. No evidence for reversibility of IDH1/2 variant-catalysed production of 2HG has been reported.

**Figure 2.**
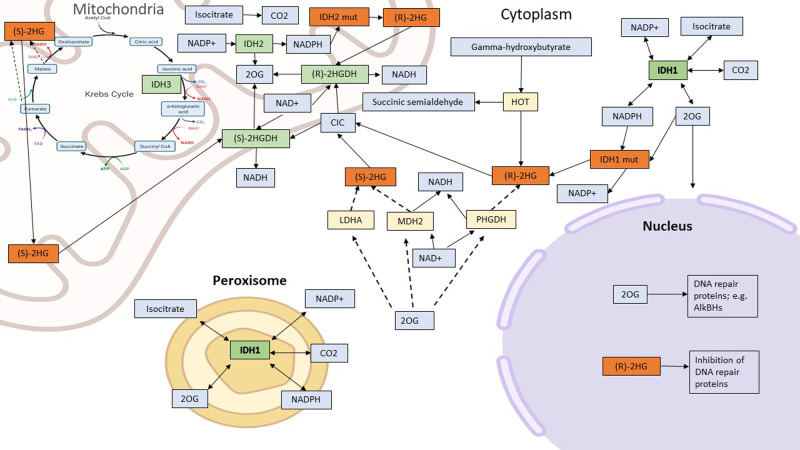
Normal functions of IDH1, IDH2 and IDH3 (green boxes) and 2HG production by variant IDH1/2 (orange boxes). IDH1 is localised in the cytosol and peroxisome; IDH2 and IDH3 localise to the mitochondrial matrix. IDH1/2 reversibly oxidise isocitrate to 2OG and CO_2_, producing NADPH. IDH3 is part of the TCA cycle and oxidises isocitrate to 2OG, producing NADH. Normal functions of IDH1, IDH2 and IDH3 (green boxes). Substrates and reaction products of normal IDH function (blue boxes). 2HG production by variant IDH1/2 (orange boxes). Solid arrows denote direct reactions, dashed arrows denote ‘promiscuous’ pathways. Note, (2*R*)-HG is biosynthesised by the metabolism of 5-hydroxy-(2*S*)-lysine, by the hydroxy acid oxoacid *trans*-hydrogenase (HOT) and phosphoglycerate dehydrogenase (PHGDH) (yellow boxes). (2*S*)-HG is biosynthesised by reactions catalysed by mitochondrial malate dehydrogenase 1 and 2 (MDH1/2) and lactate dehydrogenase A (LDHA). (*R)-*2HG and (*S*)-2HG are oxidised to 2OG by (*R*)-2HG and (*S*)-2HG dehydrogenases ((*R)*- or (*S*)-2-HGDH) (green) in reactions where an acceptor (R) is reduced (RH_2_). Abbreviations: 2OG (2-oxoglutarate), citrate transport protein (CTP), AlkB homologues (AlkBHs).

There are 3 forms of homodimeric IDH in mammalian cells. IDH1/2 (EC code: 1.1.1.42 and IDH3 (EC code: 1.1.1.41). Two of these (IDH1/2) catalyse the same metabolic reaction, namely the reversible conversion of isocitrate to alpha-ketoglutarate (α-KG) or 2-oxoglutarate (2OG), and NADP^+^ to NADPH (Figure 1). IDH1 localises in the cytoplasm and peroxisomes, whereas IDH2 and IDH3 localise in mitochondria. IDH1 and IDH2 are important for their contribution in maintaining local and cellular NADPH levels and the cellular redox balance. IDH3 also converts isocitrate to 2OG in conjunction with conversion of NAD^+^ to NADH. The IDH3 reaction is an essential and a rate-limiting step in the mitochondrial tricarboxylic acid (TCA) cycle (Figure 2). The IDH1/2, but not IDH3, reactions are reversible, with the direction apparently being determined by substrate/product concentrations [5].

Although the IDH enzymes have long been studied, aspects of their regulation are poorly understood. For example, there is evidence that wtIDH1 binds mRNA in embryonic stem cells (ESC), in particular guanine/adenine (GA) and adenine/uracil (AU) rich single-stranded mRNA, but not double-stranded RNA or DNA [6]. A significant decrease in RNA binding is reported for the R132H IDH1 variant. The function of RNA binding to IDH1 is unknown, and may be involved in *IDH1* regulation.

The IDH isoenzymes all catalyse the production of 2OG, which can also be obtained from the diet and transported into cells [7,8]. IDH1/2 catalysed reductive carboxylation can also occur leading to the formation of isocitrate and subsequently citrate from 2OG, a process which links 2OG with lipid metabolism via isocitrate production [9].

2OG is involved in a variety of metabolic processes, including the biosynthesis of amino acids and as a co-substrate for the large family of 2OG/Fe (II)-dependent dioxygenases, of which there are 60–70 in humans. The 2OG dioxygenases have diverse roles that include collagen biosynthesis, lipid metabolism, transcription, DNA repair, and hypoxia signalling [10–12]. It has been proposed that elevated 2HG levels in IDH mutant-bearing cells leads to inhibition of 2OG oxygenases involved in chromatin modification in accordance with a role for elevated 2HG in tumorigenesis [13]. It is also possible that non-enzymatic or promiscuous oxidation of 2HG to 2OG may occur with an apparently paradoxical increase in activity of some dioxygenases [14].

2HG is produced at low levels in normal (wtIDH) mammalian cells under physiological conditions by a number of enzymes (Figure 2), at least in some cases by apparently ‘promiscuous’ activities [15]. Such enzymes include phosphoglycerate dehydrogenase [16] and hydroxyacid-oxoacid transferase (HOT), both part of γ-hydroxybutyrate metabolism [17], which can produce (2*R*)-HG. The promiscuous reactions of lactate dehydrogenase (LD), pyruvate dehydrogenase (PD) and fumarate hydratase (FH), produce (2*S*)-HG [18] under acidic conditions, which is often observed in tumour and stem cells [19].

Under normal cellular conditions, cytoplasmic 2HG levels are kept low by the citrate transporter protein (CTP or CIC), which is encoded for by the *SLC25A1* gene, and which transports (2*R*)-and (2*S*)-2HG into mitochondria, where (2*R*) and (2*S*) enantio-selective NAD^+^- dependent 2-hydroxyglutarate dehydrogenases (2-HGDH) convert them into 2OG [20]. Inborn errors of metabolism arising from mutations to the genes for either of the 2-HGDH isoenzymes or CTP, can lead to accumulation of either 2HG enantiomer resulting in 2-hydroxyglutarate aciduria, with a possible increase in tumour progression [21] with associated poor patient outcomes.[22] Mosaic *IDH* mutations are characteristic of Ollier and Mafucci syndrome, which manifests raised tissue 2HG and osteoid tumours, and of specific IDH mutations, which are associated with increased risk of glioma formation [23].

Most mutations, occurring via non-synonymous single nucleotide polymorphism (NS-SNP) and other nucleotide derangements, resulting in protein variants associated with cancer, are believed to result in a ‘loss of function’ (LOF; hyper- or hypo-morphic) [24]. In the case of metabolic enzymes, loss of function is normally manifest in a failure of catalysis, e.g. to produce a specific metabolite often leading to accumulation of precursors. ‘Gain of function’ (GOF) mutations can lead to the relevant enzyme producing an alternative metabolite; in most cases, GOF is likely more difficult to identify than LOF, but the available evidence is that GOF is much rarer than LOF. *IDH* mutations have therefore attracted considerable interest because of the clear consequential GOF with the implication that the novel production and accumulation of 2HG supports tumorigenesis. The precise mechanism(s) by which elevated 2HG promotes cancer emergence and progression remain unclear. The study of elevated 2HG on cancer cell function has been extensive, for example investigation of gene expression/genomics [25], DNA repair [26], epigenetics [13], lipidomics [27], and small-molecule metabolism [28].

The canonical *IDH1 & 2* mutations (e.g. resulting in IDH1R132 and IDH2R172) are found close to intron/exon interfaces (Table 1), which typically have been found to have a lower incidence of mutation when compared with more central exon components [29].

**Table 1:** Chromosomal locations of Human *IDH1&2*, with common codon changes, exon, and proximity to the intron/exon boundary

	IDH1 R100	IDH1 R132	IDH2 R140	IDH2 R172
Codon	CGG	CGT	No data available?	AGG
Chromosome	2q34	2q34	15q26.1	15q26.1
Exon	4	4	4	4
Exon/Intron interface	No	Yes	Yes	Yes
SNP reference NCBI gene	rs276606870	rs121913500	Rs121913502	rs121913503

SNP, single nucleotide polymorphism; NCBI, National Center for Biotechnology Information.

**Table 2:** Common and rare codons with nucleotide *substitutions* corresponding to residues R132 (IDH1), R172 (IDH2), and R140 (IDH2)

Cancer type	IDH1 R132 (CGT)		IDH2 R172 (AGG)		IDH2 R140 (CGG)		Reference
	Common	Rare	Common	Rare	Common	Rare	
Glioma	CAT (H)	TGT(C), AGT(S), GGT(G), CTT(L), GTT(V)	-	AAG(K), GGG(G), ATG(M), TGG(W)	-	TGG(W)	[3,30–33]
Chondrosarcoma	TGT(C)	GGT(G), CAT(H), CTT(L), AGT(S)	S	AAG(K), GGG(G)	-	CAG(Q) TGG(W)	[34–46]
Acute myeloid leukaemia	CAT(H), TGT(C)	AGT(S)	-	-	(CAG)Q	K, TGG(W), CTG(L)	[37–41]
Intrahepatic cholangiocarcinoma	C	AGT(S), CTT(L), GGT(G)	AAG(K), TGG(W)	GGG(G), ATG(M), AAT(N)*	-	-	[42,43]
Angioimmunoblastic T-cell lymphoma	-	-	AAG(K),	GGG(G) TAT(T)*	-	GGG(G)	[44]
Sinonasal undifferentiated carcinoma	-	-	AGT(S)*	TAT(T)*, ATG(M) GGG(G),	-	-	[45,46]
Solid papillary carcinoma with reverse polarity P	-	-	GGG(G) AGT(S) TAT(T)	-	-	-	[47,48]

Encoded amino acid residues are in parentheses.

Chromosomal translocation of a gene is the most common genome abnormality associated with cancer and can alter expression levels and function [85]. Oligodendroglioma, a primary brain tumour, is commonly associated with *IDH* (1 more commonly than 2) and 1p/19q codeletion. Cancer related chromosomal translocations/deletions involving Chromosome region 2q34 (location of *IDH1*) and Chromosome region 15q26.1 (location of *IDH2*) are relatively rare. Chromosome deletions, associated with Myelodysplasia, are reported for *IDH2* Chr 15q26.1 [86], but none have been reported for *IDH1* Chr 2q34. *IDH1 or 2* deletion would be expected to be associated with loss of IDH function causing perturbed metabolism, due to a reduction in cellular 2OG and/or NADPH, with a possible decrease in the efficiency of DNA damage repair and consequently increased risk of cancer.

## Clinical incidence of IDH1, IDH2 and IDH3 variants

The distribution of *IDH* mutations in human pathology varies with the tissue of origin (Table 2). Low grade glioma has a higher incidence of *IDH 1* compared with *IDH2* mutations, of which the *IDH* R132H mutation dominates (R132H (CAT) 92.7%, R132C (TGT) 3.6%, R132S (AGT) 1.8%, R132G (GGT) 0.9%, R132L (CTT) 0.9%, R132V (GTT) 0.5%) [30]. Anaplastic R132H glioma typically present at a younger age; a trend that is not significant in rarer R132H glioma cell types. In glioma, non-canonical (i.e. not IDH1 R132H or IDH2 R170C) IDH1 variants have different clinical characteristics and tend to arise in different locations in the brain compared with the canonical variants [31]. There appears to be an even distribution of IDH1&2 mutations at low levels (less than 10%) in human cancers apart from high levels (80%) of IDH1 mutations in human low grade glioma. IDH2 mutations are more common in Acute myeloid and B-cell acute lymphoblastic leukaemia (20–33%), and cartilaginous bone tumours such as giant cell tumour of bone/osteoclastoma (80%) and osteosarcoma (28%). Within breast cancer (IDH1 mutation at 0.2%) the exception is the solid papillary carcinoma with reverse polarity (IDH2 >77%) (Table 3). In AML, *IDH2* mutations are more common than those of *IDH1* (Table 3) [38]. Interestingly, in AML, *IDH2* mutations and 2OG oxygenase Ten/Eleven Translocation enzyme (*TET2)* mutations (usually LOF) appear to be mutually exclusive, but the reasons for this are unknown [41]. Intrahepatic cholangiosarcoma have a high incidence of *IDH1* mutations as do chondrosarcomas [42], with giant cell tumour/osteoclastoma dominated by *IDH2* mutations [68]. *IDH* mutations are generally rare in some common cancers (breast, prostate and gastric), apart from some rare sub-types such as Breast Solid Papillary carcinoma with reverse polarity, where 77% of cases have *IDH2* mutations [47].

**Table 3:** Reported occurrence frequency (%) of the canonical *IDH1* and *IDH2* variants in cancers and benign tumours

Cancer type	Reported occurrence (%)			Source
	mtIDH1 (R132)	mtIDH2 (R172 or R140)	Non-canonical mtIDH1 or 2	
Central nervous system neoplasm				
Low grade glioma (grade II-III)	>70	5	0.3–2.3	[30–33]
Secondary GBM (grade IV)	55–88	3.4	-	[49]
Primary GBM (grade IV)	5–14	0.5	-	[50]
Myeloid and lymphoid neoplasms				
Acute myeloid leukaemia	6–13	8–20	0.6	[37–41,51,52,122–124]
B-cell acute lymphoblastic leukaemia	1.7	-	-	[53]
Angioimmunoblastic T-cell lymphoma	-	20–33	-	[44]
Peripheral T-cell lymphoma	-	<5	-	[54]
Myelodysplastic syndrome	<4	<4	-	[52]
Myeloproliferative neoplasm — chronic- or fibrotic-phase	<3	<1.5	-	[55]
Myeloproliferative neoplasm — blast-phase	5–12	2–9	-	[56]
Paediatric acute myeloid and lymphoblastic leukaemia	<1.5	<2.5	-	[57,58]
Bile duct neoplasms				
Intrahepatic cholangiocarcinoma	6.5–32	1–9	0.3	[42,43,59–62]
Extrahepatic cholangiocarcinoma/Clear cell extrahepatic cholangiocarcinoma	0–10	<4	-	[63]
Cartilage and bone neoplasms				
Chondrosarcoma	12–54	5–16	-	[36,64–67]
Giant-cell tumour of the bone/Osteoclastoma	-	80	25	[68]
Osteosarcoma	-	28	-	[69]
ESFT	3.3	3.3	-	[70]
Ollier disease and Mafucci syndrome related neoplasms				
Ollier and Mafucci related enchondroma and chondrosarcomas	>80	3		[71,72]
Mafucci syndrome related haemangioma	1 reported case	-	-	[72]
Mafucci syndrome related spindle cell haemangioma	70	-	-	[71]
Other neoplasms				
Breast cancer (other)	0.2	-	-	[73]
Solid papillary carcinoma with reverse polarity — rare breast cancer subtype	-	>77	-	[38,47]
Gastric adenocarcinoma	2.7	-	-	[74]
Irritable bowel syndrome-associated intestinal adenocarcinoma	13	-	-	[75]
Melanoma metastasis	1.3	-	-	[76]
Non-small cell lung cancer	0.6	0.4	-	[77]
Paraganglioma	1.5	-	-	[78]
Prostate cancer	0.3–2.7	-	-	[67,79]
Sinonasal undifferentiated carcinoma	-	35–80	-	[80]
Spindle cell haemangioma	28	7.1	3.6	[71,81]
Thyroid cancer	-	-	8–16	[82,83]
Wilms tumour	-	-	10	[84]

## IDH wild-type and variant enzyme kinetics

wtIDH1 kinetics have been studied for some time, with more recent analysis on recombinant wtIDH and its variants [87,88]. wtIDH2 and wtIDH3 have been relatively little studied from a kinetic perspective, at least using isolated recombinant enzymes. Some (at least) of the clinical IDH1 variants, including R132H, catalyse isocitrate oxidation, though like the variant catalysed reduction in 2OG to 2HG, this is a much lower rate than wtIDH1, as judged by *k*_cat_/*K*_M_ values [19]. R132H IDH1 has a high 2OG *K*_M_ of >500 µM and the *k*_cat_/*K*_M_ of R132H IDH1 for 2OG reduction is >1000 times lower than that of wtIDH1 for isocitrate oxidation. However, this is only slightly higher than that for R132H IDH1 catalysed isocitrate oxidation. The forward reaction of wtIDH1 requires Mg^2+^ (Mn^2+^ also works, but is less likely to be biologically relevant) and is inhibited by Ca^2+^ [87]. Evidence from *in vivo* studies suggests that there is sufficient free Mg^2+^/Mg^2+^ homeostasis to enable efficient wtIDH catalysis [89,90] and levels of free Ca^2+^ in cells are kept low and presumably are at insufficient levels to cause IDH1/2 inhibition, though localised effects cannot be ruled out [91]. wtIDH1 (and likely variant) enzyme kinetics are complicated. wtIDH1, like all studied clinically relevant variants, is predominantly dimeric in solution; its conformation interconverts between open and closed forms with its turnover number decreasing at high enzyme concentrations [87,88]. At least in purified recombinant form, human wtIDH1 co-purifies with two molecules of NADPH; however, its catalytically active dimeric form is reported to have half-site reactivity [87,88]. Binding of Mg^2+^ and isocitrate to its active site promotes release of one molecule of NADPH to provide a dimer with a single bound molecule of NADPH [88].

The forward wtIDH1 reaction, i.e. of isocitrate to 2OG and CO_2_, is usually studied at neutral pH, with the reverse reductive decarboxylation reaction being preferred at acidic pH [19]. The *k*_cat_ for wtIDH catalysed isocitrate oxidation increases from 20.0 ± 0.4 s^−1^ to 38.3 ± 0.9 s^−1^ as the pH increases from pH 6.2 to pH 8 with maximal efficiency about pH 7.5, (1.4 ± 0.1) × 10^3^ mM**^−^**^1^ s**^−^**^1^ [92]. These observations suggest that cellular pH may influence wtIDH/IDH variant catalysis.

Modulation of the ionisation state of Asp273 is proposed to be involved in the pH-mediated regulation of wtIDH; substitutions of Asp273 reduce catalytic efficiency and cause loss of pH regulatory effects [92]. The intracellular pH (pHi) has been measured as 7.01 ± 0.2 in normal brain *in vivo* by ^31^P Magnetic resonance spectroscopy (MRS) but is more basic (pHi 7.18) in glioma [32,93]. Combined, these observations suggest that, at least in human glioma with respect to pHi, the forward reaction of wtIDH1 is likely preferred over the reverse reaction.

## Inhibition of 2HG-producing IDH1/2 variants in cancer

As reviewed elsewhere, medicinal chemistry efforts focused on IDH1/2 variants have produced inhibitors that have been approved for clinical use or which are currently in development [94]. Interestingly, despite substantial structural variations and (in many cases) their selectivity for R132H (and/or other variants) over wtIDH1, most reported potent R132H IDH1 inhibitors, e.g. BAY-1436032 [95], GSK321 [96] IDH305 [97], and ML309 [98] (IC_50_ all <100 nM) do not appear to bind in the same active site location as do isocitrate /Mg^2+^, which is where most clinically relevant IDH1/2 substitutions occur (as evidenced by crystallographic analyses) [99]. Instead, although kinetic analyses with the inhibitors can manifest apparent substrate/Mg^2+^ competition, they bind at the dimer interface, i.e. they are allosteric inhibitors [96]. The observations of allosteric inhibition may in part reflect as yet unidentified allosteric mechanisms of IDH1/2 regulation *in vivo*. Further detailed studies on molecular aspects of the mechanisms of inhibition and IDH catalysis may aid in the development of improved IDH targeted therapies.

The development of IDH1/2 variant inhibitors has been driven by the hypothesis that reducing levels of the ‘oncometabolite’ 2HG may reduce tumour progression, halt malignant transformation, and/or improve patient survival. Several clinical trials have been performed and others are underway [100–111]. Treatment with first-generation IDH variant inhibitors results in a reduction in tissue 2HG levels [112]. However, it has been noted that inhibitor treatment did not slow tumour growth in some cases, e.g. in the case of some glioma and chondrosarcoma model cell lines [113]

The use of IDH variant inhibitors in therapy-resistant AML has shown improved survival [114], but has revealed variable impacts on plasma 2HG concentrations [115]. The successful reduction in plasma 2HG levels has been associated with ‘differentiation syndrome’ whereby immature macrophages mature and differentiate, releasing inflammatory compounds and producing a septic shock-type clinical outcome [116].

Inhibitor resistant *IDH* ‘double’ mutations have been reported in association with inhibitor treatment, with consequent re-emergence of high 2HG plasma levels [117]. IDH2 double or secondary mutations were reported to have either: (i) been present before inhibitor therapy, but present only in a small population and hence were not detected, or (ii) developed during treatment *in trans* to the canonical IDH1 R140Q gene resulting in double R140Q/Q316E and/or R140Q/I319M variants. [117]. Cell culture studies in murine Ba/F3 haemopoietic cells indicate that the ‘secondary’ mutations do not alter the efficiency of 2HG production, but interfere with allosteric inhibitor binding [117]. Isoform switching, whereby the mutation sequence switches from one IDH isoform to another (e.g. IDH1 to IDH2) has been observed after an initial response to a specific IDH inhibitor [118]. Thus, treatment with Ivosidenib, which is selective for inhibition of IDH1 variants, can result in selection for an *IDH2* mutation with consequent production of 2HG, the IDH2 variant responds to treatment with the IDH2 variant-selective inhibitor Enasidenib [119]. IDH variant drug resistance is also known to be caused by mutations in genes other than those which encode the IDHs, such as for receptor tyrosine kinase (RTK) for example [114].

## Perspectives

- *IDH* mutations are rare gain-of-function mutations that cause accumulation of high levels of intra- and extra-cellular 2-hydroxyglutarate, now considered an ‘oncometabolite’. Robust and selective inhibition of IDH1/2 variants, resulting in reduced 2HG levels, has been demonstrated and is being explored as a new avenue in cancer therapy.
- Although there are proposed mechanisms for how 2HG acts as an ‘oncometabolite’, e.g. inhibition of chromatin modifying 2OG oxygenases by 2HG, further work is required to define and validate the proposed molecular links between IDH mutations and the processes of tumorigenesis leading to cancer and progression. Exploring the potential pro-oncogenic roles of IDH1/2 variant-induced metabolic changes beyond elevated 2HG is of significant interest. The development of a molecular understanding of how changes in metabolism promotes cancer should help in the clinical deployment of IDH variant inhibitors, which have shown clear efficacy in reducing 2HG levels, but which have not always led to patient benefit.
- To circumvent the problems of emerging resistance due to doubly substituted IDH variants, the development of new generation IDH variant inhibitors, that inhibit not only the range of canonical IDH1/2 variants with a single substitution but which also inhibit the double variants, is of interest [120]. The discovery of the gain-of-function IDH1/2 variants raises therapeutic possibilities outside of IDH variant inhibition, e.g. the apparently important role of glutamine in IDH mutant tumours provides the potential for other therapeutic approaches, such as inhibition of glutaminase in combination with radiotherapy of astrocytoma as well as the possibility of combination therapies [121].

## Open Access

Open access for this article was enabled by the participation of University of Oxford in an all-inclusive *Read & Publish* pilot with Portland Press and the Biochemical Society under a transformative agreement with JISC.

## Abbreviations

AML: acute myeloid leukaemia
CTP: citrate transporter protein
GBM: glioblastoma
GOF: gain of function
HOT: hydroxyacid-oxoacid transferase
*IDH*: *isocitrate dehydrogenase*
LOF: loss of function
SNP: single nucleotide polymorphism
TCA: tricarboxylic acid
